# Germinal Center Reaction Following Cutaneous Dengue Virus Infection in Immune-Competent Mice

**DOI:** 10.3389/fimmu.2015.00188

**Published:** 2015-04-24

**Authors:** Juan Carlos Yam-Puc, Julio García-Cordero, Juana Calderón-Amador, Luis Donis-Maturano, Leticia Cedillo-Barrón, Leopoldo Flores-Romo

**Affiliations:** ^1^Department of Cell Biology, Center for Advanced Research, National Polytechnic Institute, Cinvestav-IPN, Mexico City, Mexico; ^2^Department of Molecular Biomedicine, Center for Advanced Research, National Polytechnic Institute, Cinvestav-IPN, Mexico City, Mexico

**Keywords:** dengue virus, *in vivo* B cell responses, germinal centers, antibodies

## Abstract

Dengue virus (DENV) has four serotypes, which can cause from asymptomatic disease to severe dengue. Heterologous secondary infections have been associated to a greater risk of potentially fatal dengue due to non-neutralizing memory antibodies (Abs), which facilitate the infection, such as anti-precursor membrane (prM) Abs, among other mechanisms. Usually, class-switched memory Abs are generated mainly through germinal centers (GCs). However, the cellular events underlying these Ab responses to DENV, especially during repeated/secondary infections, have been poorly studied. We wanted to know whether there is involvement of GC reactions during cutaneous DENV infection and whether there is any sort of preferential Ab responses to defined viral proteins. Intradermal DENV inoculation at a relatively low dose efficiently infects immune-competent BALB/c mice, inducing higher quantities of DENV-specific GC B cells and larger GCs than the control conditions. Interestingly, GCs exhibited as much prM as envelope (E) and non-structural 3 viral proteins *in situ*. Intriguingly, despite the much larger abundance of E protein than of prM protein in the virions, infected animals showed similar amounts of circulating Abs and Ag-specific GC B cells both for prM and for E proteins, even significantly higher for prM. To the best of our knowledge, there are no reports of the GC responses during DENV infection. This relatively stronger anti-prM response could be triggered by DENV to preferentially promote Abs against certain viral proteins, which might favor infections by facilitating DENV invasion of host cells. It is thus conceivably that DENV might have evolved to induce this kind of Ab responses.

## Introduction

Dengue virus (DENV) is one of the most significant human viral pathogens transmitted by mosquitoes and causes every year between 50 and 100 million infections worldwide, resulting in approximately 500,000 people with severe dengue (SD). It is estimated that over 40% of the world’s population is now at risk of infection. Dengue is caused by four serotypes (DENV1-4) circulating in tropical and subtropical regions of the world. It is believed that the vast majority of dengue infections are asymptomatic; however, a proportion manifests a non-specific febrile illness or progresses to classical dengue fever, characterized by fever and severe joint pain. Some of those infections can evolve to SD, dengue haemorrhagic fever (DHF), or dengue shock syndrome (DSS) (1). Various mechanisms have been associated with SD disease, highlighting among them the heterologous secondary infection, due to pre-existing, sub-, or non-neutralizing memory antibodies (Abs) (2). Although this hypothesis was proposed decades ago, it is only recently that detailed experimental proofs were provided in humans. Researchers finally unraveled that the so called facilitating Abs are directed mainly against the precursor membrane (prM) protein (3, 4). Interestingly, the prM is abundant on immature and non-infectious virions, but not in the mature particles (5–7).

Dengue virus contains three structural proteins, capsid, envelope (E), and membrane. Membrane protein is first formed as a precursor called prM (8, 9). The maturation process of DENV is directed by the proteolytic cleavage of the prM, producing then totally infectious particles (7, 10, 11). However, this mechanism is not completely efficient, and fully immature or partially mature virions are produced by host cells (5). Immature status depends on the prM cleavage, modifying size and morphology of the particles. At least 30–40% of DENV particles released from infected mosquito cells are immature, containing different quantities of prM (6). In the presence of non-neutralizing anti-prM Abs, even immature and non-infectious virus can enter the cells via Fc gamma receptors (FcγR) and replicate efficiently, leading to more infected cells, potentially contributing to a more severe disease (12, 13). On the other hand, E protein has three domains (EDI-III) (14), and it is known that EDIII is involved in virus attachment to host cell surface (15). Now, it is also known that neutralizing Abs are preferentially directed to EDIII, and recent findings have shown that Abs to EDI or EDII could facilitate DENV infection when present at sub-neutralizing concentrations (16, 17). Likewise, Abs to structural protein E can also behave as facilitating ones by enhancing infectivity of immature or partially mature particles due to recognition of epitopes that are exposed in immature virions (18, 19). In addition to the potential facilitating effects of these Abs, those that are indeed neutralizers seem to be directed against complex conformational epitopes, which are apparently expressed only when proteins are already assembled on a mature virus particle, therefore it has been complicated to dissect the precise antigenic nature of these structures (20).

Regarding the cell biology of Ab production, it is known that Abs, which had matured their affinity and had changed their isotype, are originated mainly through germinal center (GC) reactions. The GC is a very complex microenvironment where clonal B cell expansion and selection occurs in response to T-cell dependent antigens (Ag). Two crucial molecular mechanisms are utilized in the GCs, somatic hypermutation, and class switch recombination. The outcome of the GC reaction is the generation of Ab-secreting cells or plasma cells (PCs), and memory B cells (MBC), thus developing both immediate as well as long-term protection against re-infections (21–23). Long-lived PCs home to bone marrow, where they find niches for survival, and secrete increased affinity Abs (24). On the other hand, MBC activation is faster thus providing rapid protection against re-exposure to potentially dangerous Ags, differentiating then to PC, generating long-lasting B cell immunity (25). However, in DENV infection, long-lived PC and MBC are apparently “responsible” for the production of infection-facilitating Abs (26–28). Released Abs by these PCs should neutralize DENV, as apparently occurs in the case of homologous reinfections. In contrast, in the case of heterologous DENV infections, pre-existing Abs can facilitate the viral invasion of host cells. Therefore, we wanted to know whether DENV would be inducing any sort of preferential Ab responses to defined DENV Ags during crucial cellular events of *in vivo* Ab generation such as the GC reaction. To ascertain if that happens, we assessed whether GC reactions ensue the cutaneous DENV inoculation in immune-competent mice. Of note, although there are some few studies using immune-competent mice (29–32), up to now the standard animal model to study DENV infection *in vivo* is mostly limited to immune-deficient mice (33), and as far as we know, no existing model yet has used the skin as route to infect *in vivo*. Therefore, we propose a model of cutaneous infection using immune-competent animals, which can normally react to and recognize the DENV Ags. We found a similar response to E, prM, and non-structural (NS) 3 DENV proteins, despite the much bigger abundance of E protein in the viral particles. In addition, the response was significantly higher for the prM than for the E viral protein. It is conceivable that DENV has evolved to trigger this kind of Ab responses to preferentially promote infection-facilitating Abs.

## Materials and Methods

### Mice and immunizations

Specific pathogen-free male BALB/c mice 6- to 8-week-old from the breeding facilities of the Center for Advanced Research, The National Polytechnic Institute (CINVESTAV-IPN) were intradermally (i.d.) injected in the inguinal area with 6 × 10^4^ plaque forming units (pfu) of active DENV. Control mice were inoculated with the same dose of UV-inactivated-DENV, or supernatant from uninfected C6/36 mosquito cell line (C6/36), or sterile endotoxin-free phosphate buffered saline (PBS). Experiments were performed in accordance with the institutional guidelines for animal care and experimentation (CINVESTAV-IPN). Two or more independent experiments were performed, and at least three mice per group were sacrificed at each selected time point (7, 14, and 28 days post-inoculation). In some experiments, mice were i.d. inoculated with ovalbumin (20 μg). For each experimental condition, mice were inoculated at day 0 and a challenge was given at day 7. For FACS analyses, animals were sacrificed at day 14.

### Preparation of viral stock

The DENV2 clinical isolate was described previously (34). C6/36 mosquito cell line was grown in minimal essential medium (MEM) supplemented with 10% fetal bovine serum (FBS) (Gibco, Carlsbad, CA, USA) at 34°C. Baby hamster kidney (BHK-21) cells were cultured at 37°C in the presence of 5% CO_2_ in MEM supplemented with 10% FBS, 1 IU/mL penicillin, 1 μg/mL streptomycin, and 2.4 ng/mL amphotericin B. Virus stock was prepared by infecting the C6/36 cell monolayer. After 48–72 h, the infected monolayers were homogenized and diluted in 40% polyethylene glycol solution in 2M NaCl (Sigma-Aldrich, St. Louis, MO, USA) and incubated at 4°C overnight. The suspension was centrifuged at 20,000 × *g* for 1 h. The viruses were then resuspended in 1/15 of the total volume in a glycine buffer (50 mM tris, 200 mM glycine, 100 mM NaCl, and 1 mM EDTA) and 1/30 of the total volume in FBS. The viruses were homogenized, aliquoted, and frozen at −80°C until use. The viral stocks were titrated using a standard plaque-forming assay technique with BHK-21 cells, as described previously (35). To use C6/36 cells as a negative control, we implemented the same culture procedure but without infecting the cells with DENV2. For DENV inactivation, virus was exposed to UV radiation (three cycles, 3 min: 254 nm; 1000 J/m^2^). Inactivation of virus infectivity was verified by plaque assay on BHK-21 cells.

### Recombinant viral proteins and production of Ag multimers

Cultures of transformed-*Escherichia coli* strain BL21 with the recombinant plasmids containing E protein (36) and NS3 protein (37) sequence from DENV2 were induced for proteins expression by the addition of 1 mM isopropyl thiogalactoside (IPTG). The inclusion bodies were prepared and fusion proteins were then purified from a 10% preparative SDS-PAGE gel, as described previously (38). For the prM protein, PCR product encoding the full-length prM protein from DENV2 NG was cloned into the plasmid pPROEX HTa. The prM sequence was amplified by PCR using the primers: (forward) 5′ (*Hin*dIII) ccggaattcttccatttaaccacacgt and 3′ (*Xho*I) aagcggccgcaatgtcattgaagg (backward) 5′. Both the plasmid and the PCR product were digested with *Hin*dIII and *Xho*I, purified, and then ligated into the corresponding restriction sites of the mentioned parental vector. The resultant plasmid was named pPROEX/prM. The recombinant plasmid directs the synthesis of DENV2 prM protein fusion followed by a histidine tag encoded by the vector. Then, the recombinant plasmid was purified from *E. coli* JM109 with the endo-free plasmids kit (Qiagen Inc., Chatsworth, CA, USA). The constructs were verified by automated sequencing. Finally, the expression of the recombinant protein was demonstrated by the presence of a protein with the predicted molecular weight of 25 kDa. Purified proteins were biotinylated using a Biotin-7-NHS kit (Roche 11 418 165 001) at 2 mg/ml DMSO, using 30 μg of biotin/1 mg of target protein. To prepare the Ag multimers, biotinylated protein was held with Streptavidin-Pacific Blue (Sav-PB) (Invitrogen S11222) in a 6:1 (recombinant protein:Sav-PB) ratio for 30 min at room temperature (rt). The Ag multimer fraction was centrifuged in a 100-kDa molecular weight cutoff Amicon Ultra filter (Millipore) (39).

### Production of antibodies specific for E, prM, and NS3 viral proteins

Rats (6- to 8-week-old) and rabbits (10-week-old/1.8 kg) were obtained from the animal facilities of CINVESTAV-IPN. Animals were immunized with four doses of 50 μg of recombinant proteins (prM-his and NS3-gst in rats; E-gst in rabbits). These immunizations were administered subcutaneously with incomplete Freund’s adjuvant. After the third immunization, sera were screened by ELISA. Polyclonal anti-sera were adsorbed on tissue from mice, titrated and used for immunohistochemistry (IHC).

### Cell suspensions of draining lymph nodes and flow cytometry analysis

At each indicated time point, the animals were sacrificed and inguinal draining lymph nodes (DLNs) were carefully dissected and collected in PBS with 0.1% of bovine serum albumin (US Biological A1310-05). Tissues were homogenized, and viability of the cells was determined by trypan blue exclusion. Cell suspensions were analyzed by flow cytometry. Abs and reactants used were: anti-CD19-APC (Rat IgG2a, BD Biosciences 557399), anti-IgD-PE (Rat IgG2a, eBioscience 12-5993-83), Peanut agglutinin lectin-FITC (PNA-FITC, Vector FL-1071), and Ag multimers (prM-Sav-PB, E-Sav-PB or NS3-Sav-PB). Cells were treated with universal blocking reagent (Biogenex HK085-5K), then incubated with Abs, PNA-FITC, and Ag multimers for 20 min at rt in the dark, and then washed. Finally, cells were fixed with 1% paraformaldehyde and analyzed in a Beckman Coulter Cyan Cytometer using Summit software. Results were analyzed with FlowJo software v7.6.5 (Tree Star).

### Immunohistochemistry

Draining lymph nodes were obtained at different time points (days 7, 14, and 28) post-inoculation and frozen in tissue freezing medium (Tissue-Tek^®^ O.C.T. Compound, Sakura^®^ Finetek). Four to six micrometers of DLN-sections were mounted on poly l-Lysine charged slides. Endogenous peroxidase was blocked with 9% of H_2_O_2_ in PBS for 30 min at 37°C. DLN sections were incubated with the primary Abs (rabbit anti-E, rat anti-prM, or rat anti-NS3) overnight at 4°C. HRP-secondary Abs (anti-Rat Jackson Labs 112-035-143, and anti-Rabbit Jackson Labs 111-036-047) were used during 1 h at rt to detect the primary bound Ab. Enzyme-linked Abs were revealed with 3,3′-diaminobenzidine/H_2_O_2_ for 10–15 min at rt. To detect GCs, DLN sections were incubated with biotinylated-PNA (Vector B-1075), followed by HRP-conjugated streptavidin (Pierce 21124). Enzyme-linked streptavidin was revealed with Vector SG (Vector SG substrate Kit SK-4700). For double immunolabeling, the slides were treated again with H_2_O_2_, after the primary staining as above, to quench the specifically bound HRP. No counterstain was performed and the slides were mounted in polymount resin. Results were recorded using Image-Pro PLUS software for the Olympus BX51 system microscope. Pictures from the DLN and non-DLN (contralateral inguinal LN) were captured and the size was analyzed by ImageJ 1.47v (National Institutes of Health, NIH, USA).

### Serum antibody measurement (ELISA)

To measure DENV Ag-specific Abs (anti-E, anti-prM) in the different experimental conditions, serum was collected at different time points post-inoculation. Anesthetized-mice were bled by cardiac puncture. Ninety-six well E.I.A./R.I.A. immunoplates (Costar 3590, Cambridge, MA, USA) were coated with Ag multimers of protein E or protein prM (1 μg/ml) overnight at 4°C in a moist chamber. After washing and blocking, serial dilutions of serum samples were incubated for 2 h at 37°C. After further washes, HRP-conjugated anti-mouse IgG (Bio Rad 170-6516) was added for 2 h at 37°C. The reaction was visualized by the addition of 2,2′-Azino-bis(3-ethylbenzothiazoline-6-sulfonic acid) diammonium salt (ABTS) substrate (Sigma-Aldrich A1888). The absorbance was measured at 405 nm in a Sunrise Tecan microplate reader, software Magellan v.3.0 (Switzerland). Relative Ab titers were calculated after plotting the optical density of each well against the serum dilution and were derived from the linear portion of the resulting curves.

### Statistical analysis

Values were expressed as mean ± SEM. Differences between mean values were analyzed for statistical significance with GraphPad Prism 5 Software (La Jolla, CA, USA), using unpaired two-tailed Student’s *t*-test, and two-way analysis of variance (ANOVA) with the Bonferroni post-test. *P*-values <0.05 were considered statistically significant (*P < 0.05; **P < 0.01; ***P < 0.001).

## Results

### DENV efficiently infects immune-competent mice via the skin

**Figure 1:**
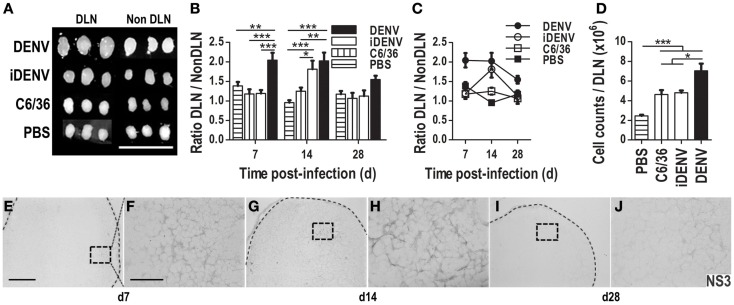
**Cutaneous inoculation of DENV infects immune-competent mice inducing an immune response**. The size of draining lymph nodes (DLN) and non-DLN (contralateral inguinal LN) was compared among groups of immune-competent BALB/c mice inoculated with either intact DENV, UV-inactivated-DENV (iDENV), supernatant from uninfected C6/36 mosquito cell line (C6/36), or endotoxin-free PBS. **(A)** DENV induces the biggest DLNs post-inoculation (14 days post-inoculation). **(B,C)** The ratio between the sizes of these LN (DLN/Non -DLN) at days 7, 14, and 28 post-inoculation. **(D)** The cellularity obtained per DLN at day 14 post-inoculation was higher in DENV inoculated mice than in mice inoculated with the other conditions. Data shown are pooled from three independent experiments, and represent the mean ± SEM. **(E–J)** NS3 viral protein was identified by means of immunohistochemistry (IHC, DAB, gray color) in DLN from DENV-infected mice at different time points post-inoculation [day 7 **(E,F)**; day 14 **(G,H)**; day 28 **(I,J)**]. Pictures in **(F,H,J)** (60×, scale bar 40 μm) correspond to the squares indicated on the left: **(E,G,I)** (10×, scale bar 200 μm). Representative pictures from one of three independent experiments are shown. Mice were inoculated i.d. with 6 × 10^4^ pfu of DENV or the other conditions at day 0, boosted at day 7, and sacrificed at the indicated days. Dotted lines depict the limits of LNs. Data were analyzed with unpaired two-tailed Student’s *t*-test, and two-way ANOVA with the Bonferroni post-test. *P < 0.05, **P < 0.01, ***P < 0.001.

To explore whether the cutaneous route can be used to infect immune-competent BALB/c mice with DENV, animals were intradermally (i.d) inoculated either with active DENV (DENV2, obtained from a clinical isolate from the National Institute for Epidemiological References INDRE, Minister of Health Mexico), with UV-inactivated DENV (iDENV), with supernatant from uninfected C6/36 mosquito cell line (C6/36, these are deemed the “gold standard” cells to easily propagate DENV), or with endotoxin-free PBS. DENV-inoculated mice showed significantly bigger DLNs than control conditions at different time points post-inoculation (Figures 1A–C). When we compared these differences by the ratio of DLN over non-DLN (contralateral inguinal LN) in these mice, DENV induced significantly larger DLNs than the control conditions. The cellularity obtained per DLN was higher in DENV-inoculated mice, confirming the findings regarding DLN size post-inoculation (Figure 1D). These results suggested that DENV is actively infecting immune-competent mice and inducing an immune response. To confirm a productive infection, we searched for the NS3 viral protein in the DLNs since it has been reported that, among the seven NS proteins, NS3 and NS5 are involved in viral RNA replication, thus their presence is indicative of viral replication (40, 41). From 7 days post-inoculation, NS3 viral protein was found in DLN, and was preferentially distributed in the cortical zone. The staining distribution for NS3 was displayed on single positive cells and in a reticular pattern inside B cell follicles (Figures 1E–J). We observed the highest intensity of NS3 staining at day 14 post-infection (Figures 1G,H). At 28 days, the labeling was scarce but still detectable (Figures 1I,J). In addition, during the first week post DENV-inoculation, mice seemed to be in discomfort, and exhibited some piloerection, altogether indicating an active DENV infection process. Pre-immune serum from non-immunized rat was used as isotype control Ab for *in situ* NS3 detection (Figure S1 in Supplementary Material).

### DENV but not the control conditions induced the largest GCs in immune-competent mice

**Figure 2:**
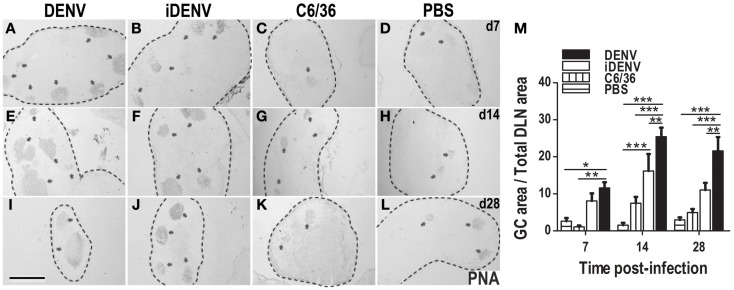
**Cutaneous DENV induces an increased number and size of GCs compared with control conditions**. **(A–L)** DLNs from mice inoculated i.d. with DENV **(A,E,I)**, iDENV **(B,F,J)**, C6/36 **(C,G,K)**, or PBS **(D,H,L)** and collected at 7 **(A–D)**, 14 **(E–H)**, and 28 **(I–L)** days post-inoculation show an increased number and bigger GCs in animals infected with DENV compared with the other conditions. The limits of LN are indicated by dotted lines. Arrowheads indicate GCs, labeled *in situ* with PNA and revealed in blue-gray color (SG Vector). Representative pictures from one of three independent experiments are shown. Mice were inoculated at day 0 and boosted at day 7. **(M)** The kinetic of GCs area as percentage of GCs Area over Total LN Area in each condition is shown. Data shown are pooled from three independent experiments, represent the mean ± SEM, and were analyzed with two-way ANOVA with the Bonferroni post-test. *P < 0.05, **P < 0.01, ***P < 0.001. Scale bar, 500 μm.

**Figure 3:**
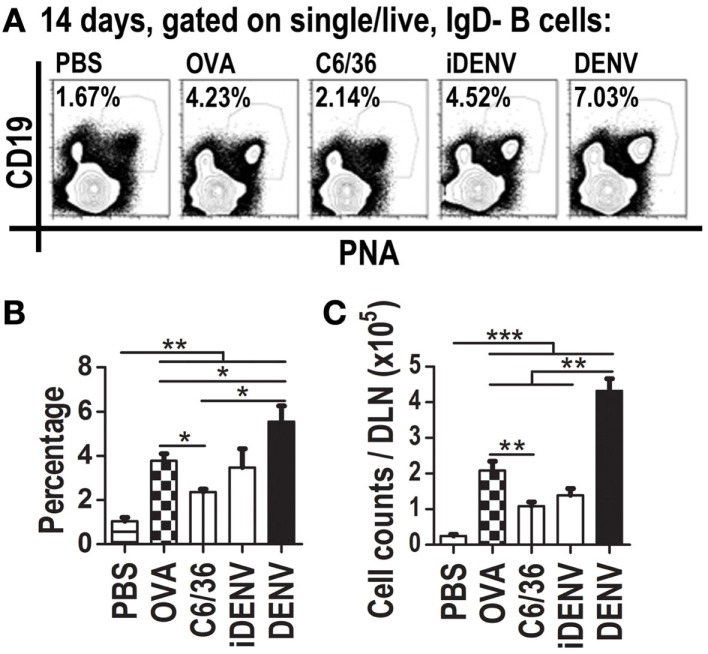
**Cutaneous DENV inoculation induces an increased quantity of GC B cells (IgD−CD19 + PNA+) in DLNs compared to control conditions**. **(A)** Representative flow cytometry plots indicate GC B cells percentage per each experimental condition described. Live IgD−singlets (not shown) were gated on GC (CD19 + PNA+) cells. Mice were i.d. inoculated with DENV, iDENV, C6/36, OVA, or PBS at day 0 and boosted at day 7. At day 14, post-inoculation cell suspensions from DLN were labeled for GC B cells. DENV inoculation induced the biggest percentage **(B)**, and the highest quantity of GC B cells per DLN **(C)**. OVA was used as positive control for GCs induction. Data shown are pooled from three to six independent experiments, represent the mean ± SEM, and were analyzed with unpaired two-tailed Student’s *t*-test. *P < 0.05, **P < 0.01, ***P < 0.001.

The presence of IgG (memory) Abs to DENV infection in humans and mice suggests that these Abs came from a GC reaction, and therefore that DENV infection is inducing GC responses. To test this, we assessed in DLNs whether GC reactions ensued cutaneous DENV inoculation. PNA labeling *in situ* within DLN showed that DENV induces a stronger GC response compared with animals receiving the different control inoculum. DENV-induced GCs were of larger number and size than GCs under control conditions such as iDENV, C6/36, or PBS (Figure 2). When we followed up the kinetics of GC area per tissue section during the infection, we found that the peak of this response was reached at day 14 post-inoculation of cutaneous DENV (Figure 2M). To quantify the number of GC B cells among the different experimental groups, we carried out flow cytometry analysis of DLN cell suspensions. GC B cells can be identified by the binding of PNA, the loss of surface IgD, and the positivity for the B cell restricted marker CD19, we thus looked for IgD−CD19 + PNA+ B cells (Figure S2 in Supplementary Material). DENV infection increased the absolute amount of GC B cells in DLN even over the GC B cells quantities induced by ovalbumin (OVA), a non-infectious Ag well-known inducer of GCs (Figure 3). The numbers of GC B cells per LN were about twice higher in DENV-inoculated mice than in OVA-inoculated mice.

### The immune response to E and to prM viral proteins is comparable in DENV-infected mice

**Figure 4:**
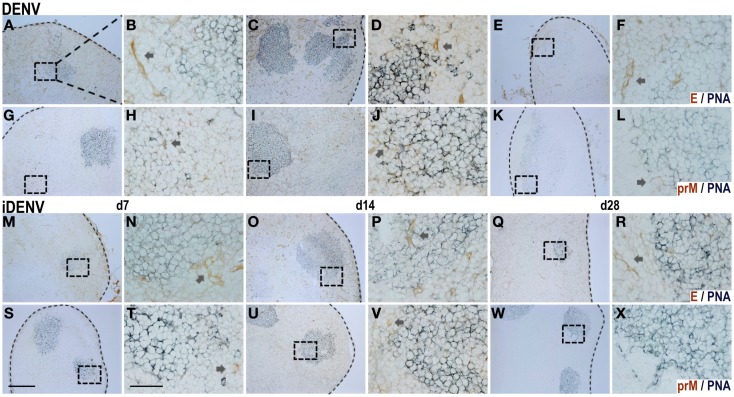
***In situ* distribution of DENV proteins inside GCs in DLN**. The viral proteins E [DENV: **(A–F)**, iDENV: **(M–R)**] and prM [DENV: **(G–L)**, iDENV: **(S–X)**; DAB, brown color] were ascertained in a concomitant double staining for GCs (PNA; SG, blue-gray color) in DLN at day 7, 14, and 28 post-cutaneous DENV-**(A–L)** and iDENV-inoculation **(M–X)**. E protein staining depicts a vascular-like pattern around GCs [arrows in **(B,D,F,N,P,R)**], unlike prM staining which shows single positive cells [arrows in **(H,J,L,T,V)**]. In the case of iDENV, the proteins distribution was scant. At day 28, the intensity of the staining diminished, and prM positive cells were fewer. For each time point, right images (60×, scale bar 40 μm, T) correspond to the squares indicated on the left (10×, scale bar 200 μm, S). Dotted lines indicate the limits of LNs. Representative pictures from one of three independent experiments are shown. Mice were inoculated at day 0 and boosted at day 7.

**Figure 5:**
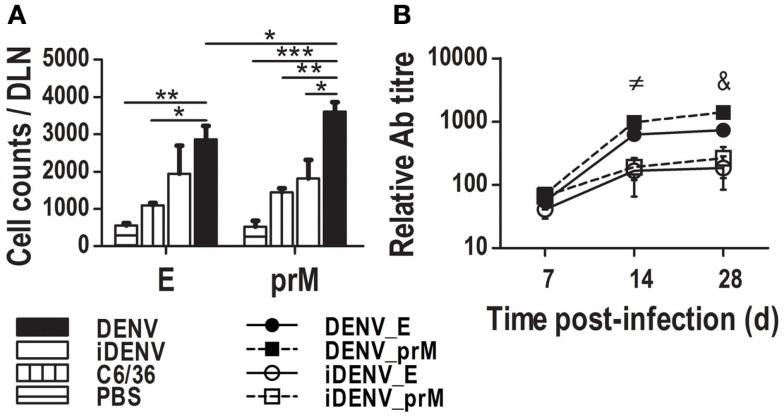
**Specific GC B cells and Ab responses to the viral proteins prM and E**. DLN from mice inoculated with different stimuli were harvested at day 14 to prepare cell suspensions. Using multimers made of prM- or E-viral proteins, we searched for Ag-specific GC B cells by flow citometry. **(A)** Total specific GC B cells per antigenic protein per LN show a higher number for prM-specific GC B cells than for E-specific GC B cells. Data shown are pooled from two independent experiments, represent the mean ± SEM, and were analyzed with two-way ANOVA with the Bonferroni post-test. *P < 0.05, **P < 0.01, ***P < 0.001. **(B)** Specific (IgG) Ab titers were determined for prM and E viral Ags, DENV induces a bigger quantity of them in comparison with iDENV. Data represent the mean ± SEM of the titers minus the titers of control C6/36, and are representative from two independent experiments. Mice were inoculated at day 0 and boosted at day 7.≠ DENV_E vs iDENV_E, and DENV_E vs iDENV_prM *P* < 0.05; DENV_prM vs iDENV_E, and DENV_prM vs iDENV_prM *P* < 0.001 & DENV_E vs iDENV_prM *P* < 0.05; DENV_E vs iDENV_E *P* < 0.01; DENV_E vs DENV_prM, DENV_prM vs iDENV_E, and DENV_prM vs iDENV_prM *P* < 0.001.

The Ab response to E viral protein in DENV infection has been studied in more detail due to their neutralizing features (42). However, although the prM Ag is also exposed on immature viral particles constituting around 40% of virions (6), the Ab response against the prM has been poorly explored despite the fact that a high proportion of the DENV infection-enhancing Abs are now known to be directed mostly to the prM (4, 16). To determine whether these GC responses could be related to these two proteins, we searched *in situ* for the E and the prM inside DLN of cutaneously inoculated mice. By IHC, we found both Ags. The positive signal for E viral protein was distributed along the whole DLN, depicting an endothelial-like structure, especially surrounding the follicles and GCs. In addition, it was possible to find the E viral protein inside GCs too (Figure 4; Figure S3 in Supplementary Material). Unlike E staining, the positive signal for the prM showed a restricted location inside B cell follicles, with some positive cells revealing a dendritic morphology. The intensity of prM labeling was comparable with that of the E protein inside the GC. In the case of iDENV, the signal for these proteins was very much weaker than that of the active DENV at the same time points assessed. Trying to support these data from another angle, we determined the amounts of prM- and E-specific GC B cells by flow cytometry using DENV Ag-multimers. We found similar amounts of E-specific GC B cells and of prM-specific GC B cells, even significantly higher for prM (Figure 5A). Furthermore, the relative titer of circulating Abs was also significantly higher for the prM protein than for the E protein in animals that were inoculated with DENV (Figure 5B). Altogether, these results suggest that the magnitude of the Ab response to prM and E viral proteins is similar, despite the much smaller amounts of prM viral protein in the virions.

### NS3 viral protein is present inside GCs

**Figure 6:**
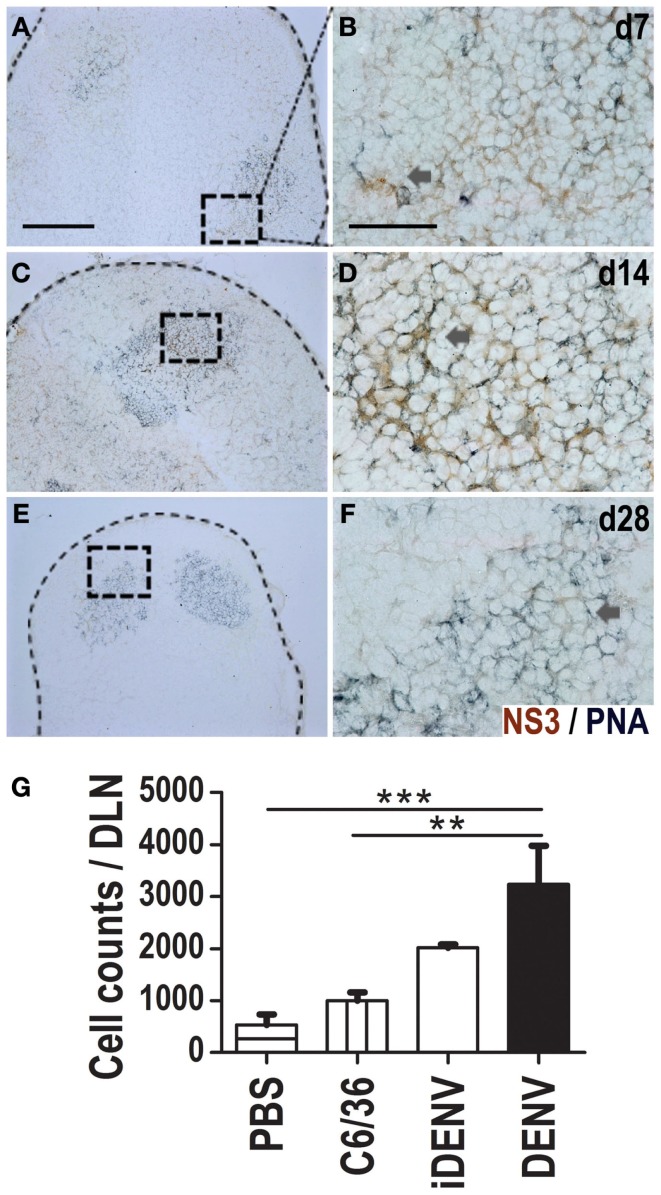
**NS3 viral protein is present inside GCs in DLNs**. Viral NS3 protein was identified *in situ* (DAB, brown color) in a double staining with GCs (PNA, blue-gray color) in DLN at day 7 **(A,B)**, 14 **(C,D)**, and 28 **(E,F)** post-DENV-inoculation. Positive cells were found inside GCs. For each time point, right images [60×, scale bar 40 μm; **(B,D,F)**] are derived from left indicated squares [10×, scale bar 200 μm; **(A,C,E)**]. Data are representative from three independent experiments. Dotted lines indicate the limits of LNs. **(G)** NS3-specific GC B cells at day 14 post-inoculation by flow cytometry using multimers of NS3 viral protein. Data shown are pooled from two independent experiments, represent the mean ± SEM, and were analyzed with two-way ANOVA with the Bonferroni post-test. **P < 0.01, ***P < 0.001. Mice were inoculated at day 0 and boosted at day 7.

Since we identified the NS3 viral protein inside DLNs of skin-infected mice (Figure 1), we wanted to know then whether we could localize this Ag precisely inside the GCs. We thus co-labeled DLN cryosections for GCs (PNA labeling) and for NS3 viral Ag. We detected NS3 inside GCs at day 7, 14, and 28 post-inoculation (Figures 6A–F). However, at 28 days the positive signal was scarce (Figures 6E,F). We detected double positive cells for NS3 and PNA *in situ*, indicative that putative GC B cells contain the NS3 Ag. Then, using flow cytometry from DLN cell suspensions, we further looked for NS3-specific GC B cells at day 14 post-inoculation (Figure 6G), we found an increased quantity of NS3-specific GC B cells in DENV-inoculated animals compared with the other conditions. Mock-infected animals (i.e., immunized with iDENV) did not show any positive signal for NS3 (Figure S4 in Supplementary Material).

## Discussion

Most animal models of diseases are developed trying to understand the mechanisms of such diseases, and therefore should be suited for studies of pathogenesis, pre-clinical testing of drugs, and vaccines, among others. Regarding DENV infection, the most used experimental models *in vivo* rely on immune-deficient mice (33, 43); however, to assess precisely the intact immune responses, these animals might not be the best indicated model. Although the mouse does not manifest dengue disease like that in humans, there are some studies done in immune-competent mice (29). These models maintain virus replication, but high doses of DENV are administered and can kill immune-competent BALB/c mice. On the other hand, DENV is inoculated by different routes other than the skin (30–32). Here, we propose an *in vivo* model in immune-competent BALB/c mice based on cutaneous inoculation, as the DENV infection occurs in nature. We were able to infect mice by intradermal injection of relatively low doses of the virus (6 × 10^4^ pfu), as this dose approximates the one that has been calculated that mosquitoes inoculate into their hosts in wildlife (44), while the reported models use higher doses of virus to cause the disease, even in immune-deficient mice (29, 33, 43). Between 7 and 28 days of DENV-skin inoculation, we found the viral protein NS3 inside DLN, indicative that the virus is indeed infecting immune-competent BALB/c mice. Although during the first week mice seemed to be in discomfort and showed some piloerection, they did not exhibit other signs of dengue-like disease. Altogether our findings suggest that at relatively low doses, immune-competent animals become infected via the skin but seem to clear the infection, and they do not present other ostensible manifestations of dengue-like disease.

The study of the potential involvement of DENV Ags in the induction and development of GCs has been rather neglected during DENV infection; and, to our knowledge, there are no reports regarding the GC response to DENV. Very few studies about DENV Ags in murine LN have been reported describing the pathology and the distribution of viral Ags; as an example, groups of mononuclear cells containing viral Ag at the periphery of the follicles have been described (29). Even for other viruses, there are few reports of Ags inside GCs, for instance, Vesicular Stomatitis Virus, Rauscher Leukemia Virus (45, 46), and a recent study with murine gammaherpesvirus 68 revealed infection of GC B cells (47). In the DENV infection in humans, the presence of viral Ags has been reported inside lymphoid organs obtained from fatal cases. Positive cells for DENV proteins have been found in blast cells inside B cell follicles, PCs, and B cells in spleen and LNs (48). The presence of positive-strand viral RNA has been related with viral replication in GC B cells (49). Specifically, NS3 viral protein has been reported in LNs, but other studies were not able to detect it (50, 51). On the other hand, it has been described that the E and the NS1 protein depict a reticular pattern inside GCs from spleen and LN (50). This pattern of DENV Ags distribution is consistent with our findings, where NS3, prM, and E viral proteins inside GCs exhibit also a reticular pattern. Our results and those of other researchers showing DENV Ags inside lymphoid tissues from both humans and mice suggest a potential relationship between these Ags and the induction of GC reactions. However, this issue has not been evaluated.

The production of memory Abs before a secondary antigenic exposure occurs constitute an evolutionary and powerful strategy to cope with subsequent infections, this may allow the neutralization of a given pathogen before the secondary infection is established (52). Memory Abs are usually generated through GC responses; nevertheless, the involvement of GCs during DENV infection is unknown and has not been assessed. By IHC and flow cytometry, we found that in immune-competent mice, active DENV is inducing larger GCs than the other experimental conditions, even larger than those triggered by a non-infectious protein, and well-known inducer of GCs such as OVA. Intact DENV can indeed infect immune-competent mice when administered via the skin at a relatively low dose, and can actively trigger the formation of GCs. Our results revealed that active DENV is a potent inducer of GC responses.

In addition to similar quantities of NS3, prM, and E viral proteins inside GCs *in situ*; by flow cytometry, we found comparable quantities of GC B cells specific for these viral Ags, even significantly higher amounts of prM-specific GC B cells than for the E protein. Data were consistent with higher quantities of Abs to prM than to E viral Ag. We think that DENV could be modulating the humoral response to preferentially promote the production of Abs against certain viral Ags. In turn, these Abs might favor a subsequent heterologous infection by facilitating the invasion of host cells, as Abs to prM have been demonstrated to enhance heterologous DENV infections (3, 4). This non-neutralizing Ab responses seem to be dominant in humans (4, 16, 17) and MBC encoding enhancing Abs predominate in the circulation, even two or more decades following DENV infection (28). The presence of the NS3 inside GCs could indicate that DENV is infecting B cells.

Although Abs to E viral protein have been described as neutralizers, it has been recently known that these Abs constitute only a small fraction of the anti-DENV binding and neutralization activity in human immune sera (4, 16, 17, 42, 53). In addition, most anti-E Abs are directed toward the EDI/EDII region and can enhance the infection at sub-neutralizing concentrations (16, 27, 28). The recent characterization of human Abs obtained by strategies to enrich neutralizing Ab-producing cells from peripheral blood mononuclear cells has made evident a variety of non-neutralizing Abs. Several of them exhibit a remarkable activity to enhance the infection (27). Moreover, Abs to all domains in the E protein can render immature DENV particles infectious in a furin-dependent manner (18). Interestingly, the most potent neutralizing serotype-specific Abs bound either to EDIII or to complex epitopes found only on the intact viral particle (20, 26, 27). Since our findings suggest that the response to prM may be similar to, or even higher than the one to the E protein, this may imply that DENV evolved to induce a mixture of Abs with facilitating features in order to enhance the probabilities of subsequent infections. These Abs could be directed to both prM and the E viral proteins.

Germinal center responses to cutaneous DENV infection in immune-competent animals might be driven by defined viral proteins which would induce non-neutralizing Abs; these Abs could then enhance the infection of other target cells. This animal model can be used to evaluate the *in vivo* humoral immune response to DENV and the different viral Ags. We consider important to understand the *in vivo* biology of these cellular responses for making better designs and public health planning in the fight against DENV infection.

## Author Contributions

Conceived and designed the experiments: JY-P, LC-B, and LF-R. Performed the experiments and the acquisition of data: JY-P, JG-C, JC-A, and LD-M. Analyzed the data, interpretation, and discussion: JY-P, LC-B, and LF-R. Wrote and revised the paper: JY-P and LF-R. Final approval of the version to be published: LF-R.

## Conflict of Interest Statement

The authors declare that the research was conducted in the absence of any commercial or financial relationships that could be construed as a potential conflict of interest.

## Supplementary Material

The Supplementary Material for this article can be found online at http://journal.frontiersin.org/article/10.3389/fimmu.2015.00188/abstract

## Abbreviations

C6/36, supernatant from uninfected C6/36 mosquito cell line; DAB, diaminobenzidine; DENV, dengue virus; DLN, draining lymph node; E, envelope; EDI-III, envelope domain I-III; i.d., intradermal; iDENV, inactivated dengue virus; IHC, immunohistochemistry; MBC, memory B cells; NS, non-structural; PNA, peanut agglutinin; prM, precursor membrane; SD, severe dengue.
