# Supplementary material for: Germinal Center Reaction Following Cutaneous Dengue Virus Infection in Immune-Competent Mice
Source: Front Immunol. 2015 Apr 24;6:188. doi: 10.3389/fimmu.2015.00188 (PMC4408864; doi:10.3389/fimmu.2015.00188)

**Germinal center reaction following cutaneous dengue virus infection in immune-competent mice**

Juan C Yam-Puc^1^, Julio García-Cordero^2^, Juana Calderón-Amador^1^, Luis Donis-Maturano^1^, Leticia Cedillo-Barrón^2^ and Leopoldo Flores-Romo^1^.

**Supporting information**

**Figure S1. Isotype control Ab for the IHC of NS3 viral protein.**

As isotype control Ab for the NS3 viral protein labelling, DLN were incubated with sera of pre-immunized rats. Labelling was revealed with DAB (brown color), there was no background staining in the tissue sections examined. Representative pictures of three independent experiments are shown. Mice were inoculated i.d. with 6 x 10^4^ pfu of DENV at day 0 and boosted at day 7. DLNs were collected at 7, 14 and 28 days post-inoculation. Dotted lines depict the limits of the LN. A-C, 10X, scale bar 200 µm.

**Figure S2. Strategy used for flow cytometry analysis.**

Dot plots show the gating strategy used to identify GC B cells. According to singlets and forward and side scatter characteristics (FSC and SSC), Ig D- cells were assessed for the expression of CD19 and positivity of PNA staining. At least 1 million cells were acquired per condition.

**Figure S3. Isotype control Abs used in IHC for E and prM viral proteins.**

As isotype control Abs for IHC, DLNs were labelled with serum of pre-immunized rabbit (for recombinant E viral protein) (A, C, 10X), and with serum of pre-immunized rat (for recombinant prM viral protein) (B, D, 10X). Labelling was revealed with DAB (brown color) and there is no background staining in tissue sections. Representative pictures of three independent experiments are shown. Mice were inoculated i.d. with 6 x 10^4^ pfu of DENV (A-B) or iDENV (C-D) at day 0 and boosted at day 7. DLNs were collected at 14 days post-inoculation. There was no background staining neither at 7 days nor at 28 days post-inoculation (data not shown). Dotted lines are limiting the LN. Scale bar 200 µm.

**Figure S4. Isotype control Abs and staining for NS3 viral protein at day 14 post iDENV-inoculation.**

DLNs were collected 14 days post-inoculation with iDENV. A. Tissue sections were stained with serum of pre-immunized rat (for recombinant NS3 viral protein). Labelling was revealed with DAB (brown color); no background staining is observed (10X). On the other hand, these LNs were stained with polyclonal serum anti-NS3 (B, 10X), revealed in brown color and simultaneously stained with PNA for GCs which was revealed in blue-gray color (C, 10X; D, 60X, scale bar 40 µm). While there was clear positive blue-gray staining for PNA *in situ* (GCs), there was not any staining (brown color) for NS3 at 14 days post-inoculation (neither at 7 and 28 days post-inoculation, data not shown). Representative pictures of two independent experiments are shown. Mice were i.d. inoculated with 6 x 10^4^ pfu of iDENV at day 0 and boosted at day 7. Dotted lines indicate the limits of LNs. 10X, scale bar 200 µm.

Figure S1


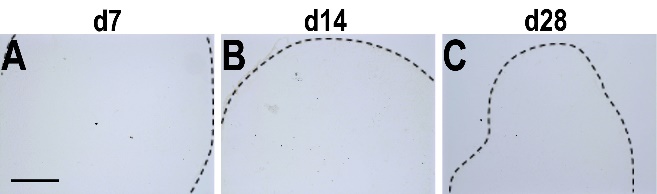


Figure S2.


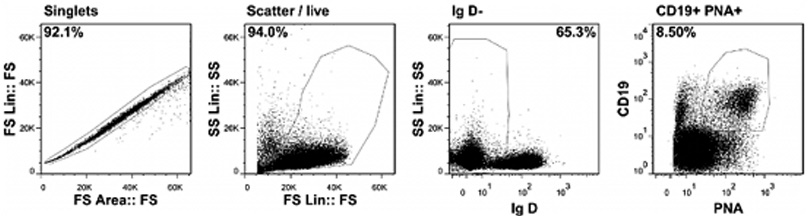


Figure S3.


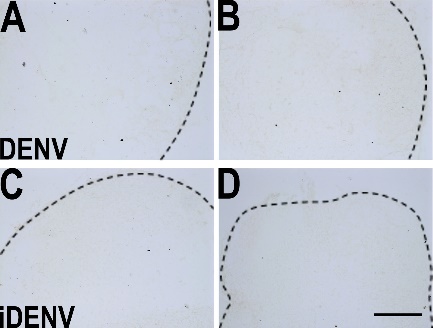


Figure S4.


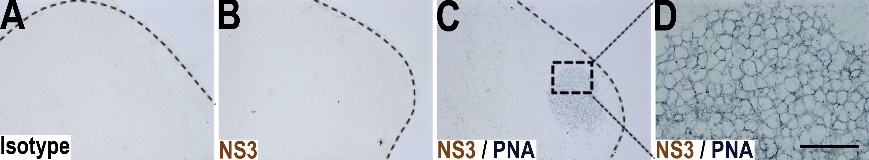

Supplement: Supplementary file 1 [file Data_Sheet_1.DOCX]
